# Developing policy recommendations for controlling energy drink consumption in secondary school students using social marketing theory, Shiraz, Iran: A study protocol

**DOI:** 10.1371/journal.pone.0321766

**Published:** 2025-04-17

**Authors:** Mohammadhassan Rostami, Mina Babashahi, Masoud Karimi, Soheila Khodakarim

**Affiliations:** 1 Student Research Committee, School of Nutrition and Food Sciences, Shiraz University of Medical Sciences, Shiraz, Iran; 2 Nutrition Research Center, School of Nutrition and Food Sciences, Shiraz University of Medical Sciences, Shiraz, Iran; 3 Department of Health Promotion, Research Center for Health Sciences, Institute of Health, School of Health, Shiraz University of Medical Sciences, Shiraz, Iran; 4 Department of Biostatistics, Shiraz University of Medical Sciences, Shiraz, Iran; Shahid Beheshti University of Medical Sciences School of Dentistry, IRAN, ISLAMIC REPUBLIC OF

## Abstract

**Background:**

Energy drink (ED) consumption has risen sharply among children and adolescents, posing health risks such as obesity and overweight, heart problems, mood disorders, and insomnia. Recognizing these concerns, international organizations have issued guidelines discouraging adolescent ED consumption, leading to policy measures in many countries. This study leverages social marketing theory to develop targeted policy recommendations for managing ED consumption in secondary school students.

**Methods:**

This study utilizes a cross-sectional design with a mixed-methods approach to collect data and formulate policy recommendations. A multistage cluster sampling method was employed to randomly select students from 24 schools, serving as the primary data source. Information is gathered through a questionnaire based on the Theory of Planned Behavior (TPB). Additionally, a food environment analysis of the selected schools, a critical factor influencing ED consumption, is conducted using the NEMS-S INFORMAS tool. This tool assesses the availability, pricing, and marketing of EDs. The study further explores stakeholder perspectives through key informant interviews and a systematic literature review, providing valuable insights into existing policy frameworks. The study aims to develop actionable policy recommendations to effectively address ED consumption by synthesizing findings from all these phases.

**Discussion:**

The social marketing model focuses on understanding the audience and evaluating outcomes to develop effective policy proposals. It is particularly useful for behavior change policies, offering evidence-based recommendations that often surpass traditional health promotion methods. This study will analyze ED consumption and its influencing factors using the model’s constructs to present informed and practical policy recommendations.

## Introduction

Energy drinks (EDs) contain high levels of sugar, caffeine, and other stimulants like guarana, taurine, and ginseng [1,2]. These beverages contain substances that act as non-nutritive stimulants and purport to have energizing or performance- and endurance-enhancing effects [3,4]. EDs were first introduced in 1960 and have been marketed to children and adolescents to increase energy, reduce fatigue, improve concentration, and mental alertness, and other uses [3,5]. Studies from Europe and the US found ED consumption in adolescents and young adults to vary between 20% and 50% [6,7]. However, there is a lack of studies examining the prevalence of ED consumption among children and adolescents in Iran.

Studies have found that consumption of EDs can cause serious side effects, particularly in children, adolescents, and young adults [8,9]. These side effects can be classified into three categories: physical, psychological, and educational.

Physically, frequent use can contribute to obesity, heart abnormalities, seizures, diabetes, insomnia, and caffeine intoxication, posing substantial health risks [6,9,10].

Psychological complications from consuming these drinks include an increased likelihood of mood and behavioral disorders, worsening depression, and even an increased risk of suicide [6,9–12]. In addition, consuming EDs during sensitive childhood and adolescence can lead to long-term negative consequences on mental health and reduce [10,11].

In the educational sphere, ED use correlates with reduced academic performance, impaired concentration, and declining mental efficiency [9,10]. Furthermore, studies highlight a strong association between frequent ED consumption and engagement in risky behaviors, such as thrill-seeking, delinquency, binge drinking, and substance use, including alcohol and tobacco [7–9].

These side effects are significant because they occur at a notable prevalence among ED consumers. For instance, around 20–30% are at risk of weight gain and metabolic disorders [13], 10–30% of users experience increased heart rate and blood pressure [5], 30% report sleep disturbances [13], and 15–20% experience anxiety and jitteriness [13], 10–20% face digestive issues [14].

Health issues like obesity, diabetes, and other non-communicable diseases in youth can have lifelong effects. Raising awareness about the risks of EDs and their impact on children’s and adolescents’ health is essential.

Given that childhood and adolescence are critical periods for physical and cognitive development, and considering the aggressive marketing targeting these age groups [15], many calls have been made globally to restrict access and sales of EDs [16]. Based on the Nutrient Profile Model for the Marketing of Food and Non-Alcoholic Beverages to Children in the Eastern Mediterranean Region (EMRO), the World Health Organization (WHO) states that the promotion, purchase, and access of EDs to children is prohibited [17]. The International Society of Sports Nutrition (ISSN) also advises that adolescents aged between 12–18 years need to be cautious while consuming EDs, particularly when the consumption exceeds 400mg. Moreover, the ISSN does not recommend the use of EDs for children aged between 2–12 years old [18]. To clarify, a typical 250ml ED can contain between 80–150mg of caffeine, depending on the brand [19].

Moreover, the WHO and the American Academy of Pediatrics (AAP) advise countries to implement strict policies to reduce the consumption of EDs among children and adolescents (under 18 years old) [20,21]. In response, several countries have introduced regulations and policies—such as fiscal measures, access restrictions, and advertising bans—aimed at transforming the food environment into a healthier one to limit ED consumption [16,22,23]. In Iran, regulations require EDs to display warning labels indicating that their consumption is not recommended for individuals with specific health conditions, pregnant or breastfeeding women, children, and those sensitive to caffeine or with high blood pressure. Other measures include prohibiting the sale and advertising of EDs in schools and imposing taxes on these products [24].

However, factors such as deficiencies in the design of regulations, conflicts of interest, and resistance from industries have contributed to the continued widespread availability, aggressive marketing, and relatively low cost of EDs. These factors – which as part of an unhealthy food environment can exacerbate its negative impact – have been significant contributors to the increased consumption of these beverages among children and adolescents [24–26], leading to a greater prevalence of their consumption in recent years [3]. This surge in ED consumption in recent years highlights the critical need to address environments where young individuals are most influenced—particularly schools.

Recognizing that schools are key environments where children and adolescents spend the majority of their time, it becomes evident that these institutions play a pivotal role in shaping dietary behaviors. The pervasive availability and aggressive marketing of EDs within and around school settings further reinforce unhealthy consumption patterns among students. Therefore, addressing the school food environment—where EDs may be readily accessible—is essential in the broader strategy to create healthier food environments [27].

This focus on schools aligns with the broader concept of the food environment, which encompasses physical, economic, political, and sociocultural factors that influence food choices and nutritional status [28,29]. A healthy school food environment, as an integral part of this larger system, not only enables but also encourages students and their families to make healthier food choices as well as nutritional knowledge. This, in turn, contributes to improved well-being and nutritional outcomes, as supported by various studies [30–33]. However, achieving such an environment requires comprehensive, multi-faceted strategies that extend beyond conventional health promotion efforts, integrating policy reforms and community engagement to ensure sustainable improvements.

In recent years, the social marketing approach has gained traction among policymakers for developing public health policies [34]. This approach is particularly suited to addressing complex issues within the food environment because it systematically identifies problems and their influencing factors. By understanding these dynamics, social marketing enables the development of targeted and actionable solutions. Unlike traditional policy-making, social marketing emphasizes consumer-focused strategies, making it a valuable tool for evaluating, planning, and implementing interventions to improve the food environment [35–37].

One of the strengths of the social marketing approach is its multi-stage process, which includes audience analysis, channel analysis, and market analysis [37]. There is evidence to suggest that this planning approach may be more effective than traditional approaches used in health promotion. This is due to its multi-stage focus on the consumer, which differs from most planning models in health promotion settings [34].

The integration of behavior change theories, such as the Theory of Planned Behavior (TPB), can be a key factor in enhancing the effectiveness of audience analysis in social marketing. TPB provides a cognitive framework for understanding and predicting health-related behaviors, focusing on attitudes, subjective norms, and perceived behavioral control [38,39]. This theory has been shown to be effective in predicting healthy eating behaviors in diverse populations and settings [40–42]. This integration strengthens the customer-focused approach of social marketing by merging its strategic planning framework with the theoretical foundations of TPB. By incorporating TPB, social marketing campaigns are not only strategically refined but also achieve a more targeted and impactful method for driving behavior change.

Based on the outlined evidence, it is clear that gathering data on the prevalence and determinants of ED consumption among children and adolescents is crucial. Given the significant influence of the school food environment on students’ dietary choices, this study focuses on examining the consumption patterns of EDs and identifying the factors driving their use among secondary school students. Utilizing the principles of the social marketing approach, the study seeks to provide actionable insights into this pressing issue.

While several countries have implemented policies to limit ED consumption among young populations, including restrictions on advertising, labeling requirements, and bans on sales in certain settings, Iran currently lacks specific legislation targeting ED consumption. Although there are broader regulations addressing foods high in sugar, salt, and trans fats, the absence of targeted policies for EDs underscores the need for a tailored approach.

To address this gap, the study will leverage the principles of the social marketing approach to develop evidence-based policy recommendations. By doing so, it aims to propose new policies or refine existing ones to better regulate ED consumption. Therefore, the purpose of this study is to use social marketing theory to develop policy recommendations for controlling ED consumption in secondary school students of Shiraz City, Iran in 2024.

## Materials and methods

Using social marketing theory, this study aims to formulate policy recommendations for managing ED consumption among secondary school students in Shiraz City, Iran in 2024. The study employs a cross-sectional design and utilizes a mixed-methods approach for data collection and analysis. The qualitative component of this study is grounded in the phenomenological paradigm, which focuses on understanding individuals’ lived experiences [43]. This paradigm seeks to explore and describe the essence of a phenomenon by examining it through the perspectives of those who have directly experienced it, aiming to uncover both the nature of the experience and its deeper meaning [44].

### Ethics approval

This study was approved by the student research committee at Shiraz University of Medical Sciences and assigned the ethics code IR.SUMS.SCHEANUT.REC.1402.165.

### Conceptual framework

#### Social marketing model.

This study uses the social marketing model to develop evidence-based and targeted strategies to control ED consumption among children and adolescents. The process begins with gathering detailed information on target behaviors, attitudes, and contextual factors to identify key policy objectives, carried out under the framework of audience analysis. Next, the study evaluates existing policies and strategies for their relevance, feasibility, and effectiveness while addressing potential barriers such as costs, stakeholder resistance, and implementation gaps, within the market analysis framework. Finally, it identifies the most effective pathways for policy implementation by analyzing the successes and limitations of existing programs and frameworks, ensuring the proposed recommendations are practical and impactful. Below, the key stages and constructs are detailed:

1. **Audience analysis**

**Fig 1:**
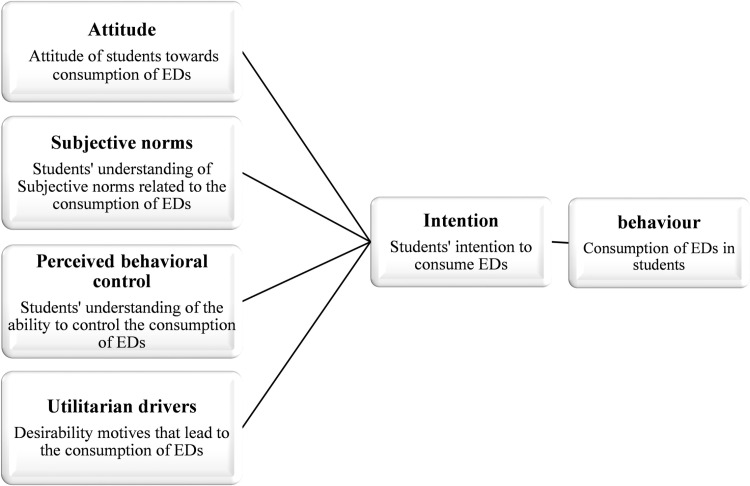
Extended version of the theory of planned behavior.

The initial step involves conducting a comprehensive assessment of the target population, with a focus on their behaviors and an evaluation of the school food environment as a critical contextual factor influencing those behaviors. This analysis is divided into two main components: Consumer Analysis and Food Environment Analysis.

a. Consumer Analysis: This component employs the extended TPB, as adapted from the study by Samoggia et al. [45]. Permission has been secured from the original authors to integrate this model into our research. The extended TPB evaluates five critical factors that shape behavior: 1)Behavioral Intention: A predictor of actual behavior 2)Attitude: The individual’s evaluation of the outcomes of behavior 3)Subjective Norms: Social pressures from significant others 4)Perceived Behavioral Control: The individual’s perception of facilitators and barriers 5)Utilitarian Drivers: Practical, functional, and satisfaction-related motivations [38,45,46]. These constructs are visualized in Fig 1.

b. Food Environment Analysis: Recognizing the profound impact of environmental factors on behavior, this study integrates the Brennan Children’s Food Environment Framework to assess the food environment within school settings. This framework examines four critical micro-environments: 1)Physical environment: Availability of EDs in or around schools 2)Economic environment: Pricing and taxation of EDs 3)Social environment: Peer, family, and teacher influence 4)Communication Environment: Marketing and advertisements targeting children [47].
2. **Market analysis**

This stage evaluates the proposed policy recommendations using the 4Ps of the marketing mix to reduce ED consumption among children and adolescents:

a. Product: In this study, “product” refers to the policy recommendations aimed at reducing ED consumption.
b. Price: This aspect encompasses all costs associated with implementing the policies, including financial, social, and political costs. Challenges such as conflicts of interest with related industries, stakeholder resistance, and the potential emergence of illegal markets are considered.
c. Place: Refers to the settings where the policies will be implemented. These settings may include schools, their surrounding environments, local stores, and other areas where children and adolescents commonly access EDs.
d. Promotion: Includes strategies to raise public awareness and encourage the adoption of the proposed policies. These strategies may involve health awareness campaigns, collaboration with health authorities, gaining the support of parents and teachers, and leveraging mass media for information dissemination and education.

1. **Channel analysis**

At this stage, existing laws and experiences from other programs and policies will be analyzed to determine which laws have been successful and in which areas. By examining these cases, the strengths and weaknesses of existing programs and policies will be analyzed to determine which channels can lead to more successful outcomes.

**Fig 2:**
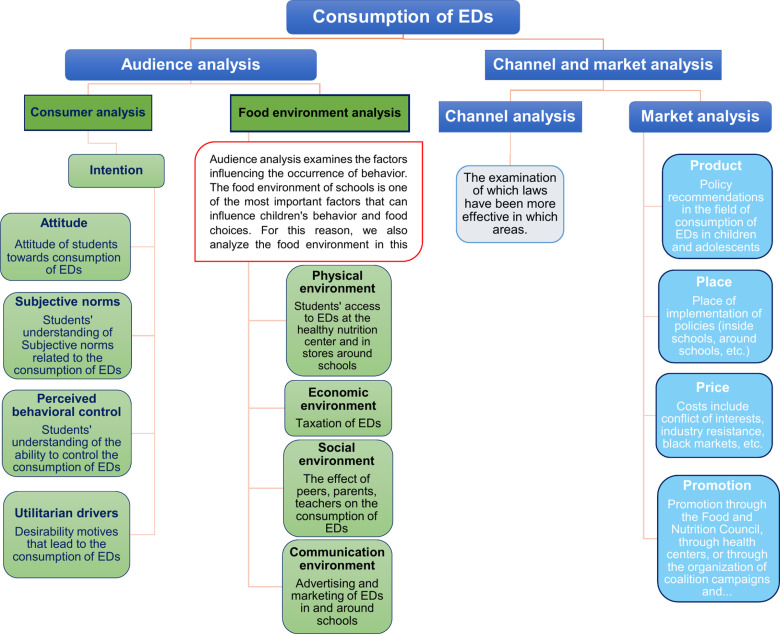
Conceptual framework.

Synthesis: In this study, we will combine all frameworks and models into a single framework, as shown in Fig 2.

### Setting

The study is planned to take place in secondary schools located in Shiraz. According to the latest report by the Shiraz Municipality Program and Budget Office, the city is divided into 11 regions, each with varying economic and social statuses. According to the 2016 census, the total population of Shiraz is 1,869,001, with 5.39% of this population consisting of adolescents aged 15–19 [48].

**Fig 3:**
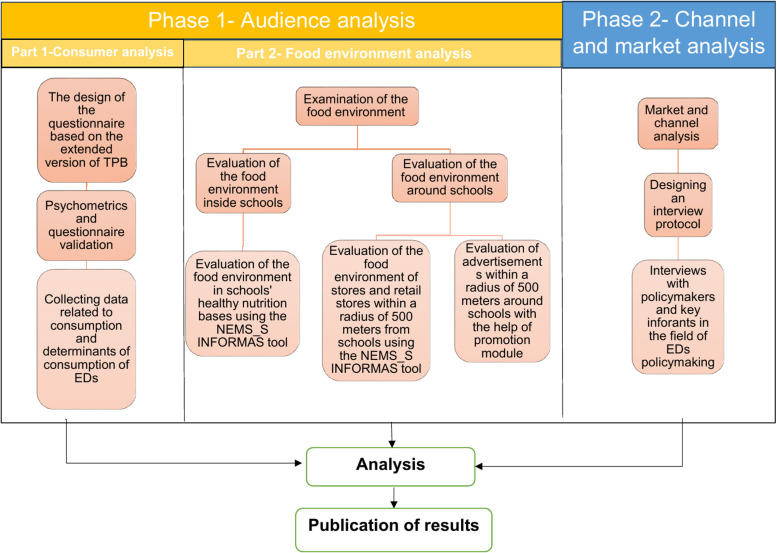
Flow chart of the study.

The study flow chart is briefly shown in Fig 3.

#### Phase 1 – audience analysis.

This phase of the study followed the guidelines outlined by the Strengthening the Reporting of Observational Studies in Epidemiology (STROBE) during the first phase [49].

***Part 1 - Consumer analysis.* Participants:** In this study, multistage cluster sampling will be used. The sample size will be calculated through $N=\frac{{\left(z_{1-\frac{\alpha }{2}}+Z_{1-\beta }\right)}^{2}p\left(1-p\right)}{d^{2}}$ formula. To know the information on the prevalence of ED consumption, the study by Almulla et al. in 2020 will be used [50].


$$N=\frac{z^{2}p\left(1-p\right)}{d^{2}}=\frac{{\left(1.96+0.84\right)}^{2}0.27\left(1-0.27\right)}{0.05^{2}}=618$$

Based on data from the Shiraz Municipality Program and Budget Office and existing studies, Shiraz is classified into 11 municipal regions, divided into three socioeconomic groups: high, medium, and low [51,52]. Within each socioeconomic group, schools are further categorized based on their type (public or private) and the gender of their students, resulting in 12 distinct clusters. From each cluster, two schools will be randomly selected, totaling 24 schools. In each school, 27 students will be selected using simple random sampling to complete informed consent forms and questionnaires, ensuring the target sample size of 618 participants is achieved.

**Inclusion criteria:** The inclusion criteria for this study will include being Iranian, aged 16–18 years, willingness to participate, not following a specific diet, and the absence of specific illnesses or conditions (i.e., conditions managed by a physician or requiring specific medications) in the students.

**Questionnaire design:** A questionnaire based on the extended TPB will be developed for consumer analysis. This questionnaire will assess the determinants, frequency, and amount of ED consumption, along with background information related to their use. Furthermore, it will examine ED consumption within the framework of the extended TPB. The design, validity, and reliability of the questionnaire will follow the comprehensive framework proposed by Tsang et al. [53]. To ensure its scientific rigor, feedback will be obtained from an expert panel comprising specialists in nutrition, school health, health education, health promotion, research methodology, and other relevant fields.

The subject domains of the questionnaire will be identified through the following approaches:

The subject domains for this study will be derived from the findings of Treloar et al. (2017), Yujia Wang et al. (2016), and Samoggia et al. (2021), which provide valuable insights into students’ intentions, attitudes, subjective norms, perceived behavioral control, and utilitarian drivers associated with ED consumption [45,54,55]. These studies will serve as a foundation for questionnaire development, ensuring alignment with established constructs. To enhance this approach, 3–4 focused group discussions, each lasting approximately 45 minutes, will be conducted to gather insights from students, the primary group in the study. These discussions will explore their views on EDs, including perceived benefits, potential harms, and social influences, such as the impact of peers and family. A comprehensive literature review will also identify additional determinants and contextual factors, ensuring the study captures a holistic understanding of ED consumption behaviors.

Data collected will be analyzed systematically using MAXQDA 2020 software. An integrative inductive-deductive approach will be employed, organizing the data into codes based on the study’s objectives. Similar codes will be grouped into overarching primary codes to ensure clarity and coherence in the analysis.

To enhance coding accuracy and reliability, two independent coders will review the data. Inter-coder reliability will be calculated using Holsti’s formula, and any disagreements will be resolved through discussion. Additionally, intra-coder reliability will be assessed by having a second coder independently code 20% of the focus group data. The intra-coder reliability coefficient will be calculated to ensure consistency in the analysis.

The iterative coding process will begin immediately after each focus group session. Based on the finalized codes, preliminary questionnaire items will be developed. The questionnaire will undergo face and content validity assessments by a panel of experts. Content validity will be evaluated using the Content Validity Ratio (CVR) and Content Validity Index (CVI), while face validity will be assessed using the Item Impact Score (IIS). Internal consistency of the questionnaire constructs will be confirmed using Cronbach’s alpha values. To ensure the questionnaire is accessible and comprehensible for the target audience, a pilot test will be conducted with a group of students, and their feedback will inform further refinements.

To confirm reliability, the test-retest method will be applied with a two-week interval on a sample of 30 students. The Intraclass Correlation Coefficient (ICC) will be calculated to ensure consistency over time. After final validation and reliability testing, the finalized version of the questionnaire will be prepared for use in the study.

***Part 2 – Food environment analysis.* Participants:** In this part of the study, EDs will be examined in school healthy nutrition centers as well as in stores and retail outlets located within a 500-meter radius of selected schools in Shiraz City. The sampling method in this stage is consistent with the first stage, involving the evaluation of healthy eating bases of schools, along with stores and retail outlets around 24 pre-selected schools. The exact locations of the stores will be identified and reported using ArcGIS software.

**Inclusion criteria.** Inclusion criteria include filling out an informed consent form and obtaining permission from the retail store owner or general manager of the chain store to collect data.

***Data collection.* Evaluation of the food environment of stores around schools and schools’ healthy eating bases:** The food environment will be assessed using the upgraded version of the NEMS-S (Nutrition Environment Measures Survey in Stores), known as NEMS-S INFORMAS. This enhanced tool combines the original NEMS-S survey with the INFORMAS retailer module, offering a more comprehensive examination of the food environment.

The researcher will visit stores within a 500-meter radius of the selected schools. Before starting the evaluation, store owners or managers will complete a consent form. Once permission is granted, the researcher will assess variables such as the prices of different food groups and items, the availability of specific food products, and the characteristics of food shelves, including their width, depth, and quantity. The evaluation will also cover food advertisements displayed inside and outside the stores and the prominence of food items in various sections of the store.

All findings will be recorded on a protocol form that outlines the study’s methodology and data collection details. The completed forms will then be immediately entered into the designated software for analysis.

**Evaluation of food advertisements around schools:** The Food Promotion Module from INFORMAS will be utilized for this evaluation within a 500-meter radius of the selected schools. This module will help assess advertising patterns and ensure they are aligned with existing food marketing policies.

The researcher will document all advertisements around the schools, including billboards, banners, posters, and other promotional materials. Using the relevant protocol form, the researcher will fill in the associated checklist to gather detailed information. Additionally, all advertisements will be photographed and the images will be attached to the protocol form for further analysis.

The data will be collected on the same day, and all information, including the photographs, will be entered into the designated software. The photos will be imported into MAXQDA version 2020, where they will be analyzed using the photo-elicitation method for coding and interpretation.

#### Phase 2 – Channel and market analysis.

This phase of the study adhered to the guidelines outlined by the Consolidated Criteria for Reporting Qualitative Research (COREQ) in the second phase [56].

In this phase, two methods will be used to collect information:

1. Literature review: The first step involves reviewing existing texts, documents, and articles related to policies and laws concerning EDs in Iran. This will include articles, books, reports, and other relevant electronic sources. Data relevant to the objective will be extracted from these materials. Keywords related to ED policies will be identified from various studies in the field, and a systematic search will be conducted in databases such as PubMed, Web of Science, and Scopus, focusing on publications from 2000 onwards. The goal is to identify and gather texts and documents concerning the existing policies and regulations regarding EDs in Iran.
2. Interview: This part of the study will focus on analyzing the channels and markets of social marketing constructs. Participants will include key informants with insights or opinions on policies and programs to reduce ED consumption. The participants will be selected from various fields, including national and subnational representatives from the Ministry of Health, Ministry of Education, and Ministry of Industry, Mine, and Trade, informant members of the Secretariat for the Supreme Council of Health and Food Security, the National Nutrition and Food Technology Research Institute, officials from the community nutrition unit at health and treatment centers, school health officials, nutritionists, and experts in food security and public health policies. Interviews will take place at the informants’ workplace, and prior to the interview, an informed consent form will be completed. Interviews will only occur if the form is filled out. Each interview will be conducted privately between the researcher and the key informant. Key informants will be selected using the snowball sampling method, where each participant will be asked to suggest other knowledgeable individuals in the field, leading to further identification of relevant informants.

***Data collection:*** Key informants will be interviewed face-to-face using the questions outlined in S1 File. These interview questions may be subject to change following the initial pilot phase, which includes conducting a few preliminary interviews to refine the process.

Participants will be contacted to schedule appointments, and the purpose of the meeting will be explained beforehand. Interviews will proceed only with the informants’ permission.

An informed consent form will be provided to participants before the interview begins. If participants are unwilling to sign the form or choose to leave the study at any stage, they will be excluded from the study. With the permission of the experts, interviews will be recorded digitally, and key points will also be noted down. If participants refuse audio recording, the interview will be manually documented in a notebook. Each interview will last approximately 45 minutes.

After the interviews, the transcriptions will be sent back to participants for their review, correction, and comments. Following each interview, the content will be carefully reviewed and coded. Both explicit content (the transcribed text) and hidden content (researcher interpretations, pauses, body language, etc.) will be analyzed. The researcher will extract relevant insights to achieve the study’s objectives.

The interview process will continue until data saturation is reached, meaning no new information is emerging from the interviews. At the end of the interview, participants will be invited to suggest any additional relevant information or points they feel should be included. The content of the interviews includes the explanation of the components of the 4Ps, i.e., policy recommendations in this area (product), the costs of changing or adopting new policies (cost), the effective location of policies and programs (location), and how to promote and market these policies and programs (advertisements). Validation of interview data will be done based on the framework proposed by Guba and Lincoln in 4 parts [57]:

1. **Credibility:** credibility means a conscious effort to ensure that the meaning of the data is interpreted in terms of accuracy and correctness.
2. **Dependability:** it is called the stability of data over time and different conditions, and it is somewhat similar to reliability in the discussion of quantitative research.
3. **Confirmability:** confirmability can be guaranteed by presenting the research process in a way that can be followed.
4. **Transferability:** transferability refers to the extent to which the findings of the study can be transferred or used in other groups or places.

### Statistical analysis

To measure validity and reliability, indexes such as content validity ratio, content validity index, and Item Impact Score will be calculated. Also, Cronbach’s alpha will be used to check the intraclass reliability coefficient (ICC) and to measure internal consistency. Also, the mean, standard deviation, median, and 25th and 75th percentiles will be used to report the descriptive statistics of quantitative variables, and frequency and percentage will be used for qualitative variables. Logistic regression will be used to determine the relationship between consumption of EDs and demographic variables. SPSS version 26 and Excel version 2021 will be used for data analysis. We will use linear regression to compare the desired indicators such as availability, price, and advertising in different economic and social areas. Quantitative variables, such as ED consumption in different socio-economic areas, are evaluated using the same test. In this study, an attempt has been made to consider potential biases in the reporting of variables such as ED consumption. Also, to determine the relationship between the consumption of EDs and the desired indicators, logistic regression will be used. In addition, to check the amount of advertisements of different food groups every 100 meters distance from schools, the Poisson distribution index will be used. SPSS version 26 will be used for data analysis.

## Discussion

Policies in the macro field should aim to change behavior, and governments need to explore new and effective tools to direct and reinforce these behaviors. While legislation and mandatory laws are commonly used as hard tools to bring about changes in behavior, they can often be ineffective and expensive. For this reason, policymakers should look for effective and less mandatory solutions and tools [34].

Social marketing is an approach to developing activities to change or stabilize a social behavior for the benefit of society [58]. It is a powerful way to influence attitude and behavior with the help of an interactive approach in providing information and using behavior change tools based on social science studies [59] and helps to facilitate acceptance, rejection, modification, abandonment, or continuation of behavior in a population group [60].

The social marketing model also can provide a unique opportunity for policymakers related to behavioral change approaches [61]. Social marketing deals with policymaking mainly in the policy formulation stage. What makes social marketing an effective tool is related to helping to analyze the desired problem and providing appropriate solutions, which can ultimately lead to appropriate and acceptable policy recommendations in the field of behavior change [61].

One of the places for implementing these policies is the food environment surrounding schools. Policies aimed at improving school food environments can positively influence children’s eating habits, ultimately helping to reduce obesity and overweight issues by creating healthier food options [62]. The school environment plays an essential role in students’ dietary patterns. Studies have shown that the variety and quality of food available around schools can influence this population’s health and nutrition outcomes [30,31]. Therefore, the food environment is one of the places that can be targeted by policymakers to control the consumption of these drinks among students by changing access, and pricing, and banning food advertisements, including EDs.

So far, no study has been conducted on the prevalence of ED consumption among secondary school students in Iran. However, due to the increasing popularity of these drinks among children and teenagers and the awareness of the side effects of consuming these drinks in this age group, it is felt necessary to conduct a study in this field. Considering that the food environment such as access, price, and advertising can play an important role in the food choices of children and teenagers, it is essential to know the state of the food environment of schools in this area. Also, due to the experience that different countries around the world have approved and implemented laws in this field, the need to know and analyze the target audience and factors affecting the choice of EDs is felt more than ever. In this study, an attempt will be made to analyze the current situation in the field of consumption of EDs by using the principles of social marketing as an efficient tool in analyzing the current situation and the potential of this model in providing evidence-informed policy recommendations and to recommend suggestions to change, Reinforce and improve existing policies or even recommend new policies.

The purpose of this study is to collect information about the state of the food environment in and around schools, as well as to analyze information related to the constructs of the extended TPB among students that can help the policymakers of this area by drawing a perspective of the current food environment of children and teenagers to improve the food environment of this vulnerable group. It is also an effort to collect information and data, relying on the principles of social marketing, to make policy recommendations to improve the health of children and adolescents and reduce healthcare costs related to the complications of consuming EDs in this group to make it available to the policymakers of this field to adopt new policies by further improving the existing policies.

### Strengths and limitations

This study has utilized established and recognized frameworks such as social marketing theory, the extended TPB, and other analytical frameworks to assess consumer behavior and the food environment. This approach has enhanced the credibility of the methodology and the study’s findings. Additionally, the use of a mixed-methods methodology, combining qualitative and quantitative approaches, has allowed for the collection of more comprehensive data. By employing tools such as interviews, questionnaires, and food environment analysis, the study has gathered richer data, contributing to a more thorough analysis of the topic. Moreover, the analysis of consumer behavior and the food environment in this study has identified factors influencing the consumption of EDs in a detailed manner. These analyses could be especially useful in shaping future policies. The use of a multi-stage sampling method, which considers the economic and social diversity of different areas of the city, has facilitated the collection of representative data that can be generalized to the target population.

The limitations of this study include several aspects. First, the use of self-reported data through questionnaires and interviews may lead to response biases. Participants may inaccurately report their behavior due to social expectations or a desire to present themselves in a more favorable light. Additionally, the evaluation of the food environment faces challenges such as the lack of cooperation from vendors and limited access to accurate data. Assessing the impact of advertisements and physical environments can also be time-consuming and complex. Furthermore, this study only evaluates the food environment around schools, while children and adolescents are also present in the home and neighborhood food environments. These environments, which can significantly affect children’s food choices, were not investigated in this study. These limitations may impact the comprehensiveness of the results and could influence future policy-making.

## Supporting information

S1 File Interview questions. (DOCX)

## Abbreviations

EDs: energy drinks
ED: energy drink
TPB: theory of planned behavior
