# Supplementary material for: Developing policy recommendations for controlling energy drink consumption in secondary school students using social marketing theory, Shiraz, Iran: A study protocol
Source: PLoS One. 2025 Apr 17;20(4):e0321766. doi: 10.1371/journal.pone.0321766 (PMC12005526; doi:10.1371/journal.pone.0321766)
Supplement: S1 File — (DOCX) [file pone.0321766.s001.docx]

**Supplemental File 1- Interview Questions**

1. What is your perspective on the current status of energy drink consumption in society, particularly among adolescents?
2. Are the government or health organizations in Iran currently taking any actions to limit energy drink consumption among adolescents? If yes, what policies have been implemented?
3. Do you think the existing laws are sufficient to control energy drink consumption, or do they need to be strengthened?
4. What factors do you believe are preventing the full implementation of current policies aimed at reducing energy drink consumption among adolescents?
5. Do you have any specific suggestions to improve the effectiveness of policies in reducing energy drink consumption among adolescents?
6. In your opinion, which places (e.g., schools, universities, or social media) would have the most impact on controlling energy drink consumption among adolescents?
7. What potential conflicts of interest should be considered, and how can they be managed?
8. Are there any additional insights or information that you believe would be useful for this study?
9. Based on the questions discussed and the topic under investigation, do you know anyone else who might be able to help us address our research questions?
